# A genome-wide association study for somatic cell score using the Illumina high-density bovine beadchip identifies several novel QTL potentially related to mastitis susceptibility

**DOI:** 10.3389/fgene.2013.00229

**Published:** 2013-11-06

**Authors:** Brian K. Meredith, Donagh P. Berry, Francis Kearney, Emma K. Finlay, Alan G. Fahey, Daniel G. Bradley, David J. Lynn

**Affiliations:** ^1^Animal and Bioscience Research Department, Teagasc, Animal and Grassland Research and Innovation CentreGrange, Dunsany, Co. Meath, Ireland; ^2^School of Agriculture and Food Science, University College DublinDublin 4, Ireland; ^3^Animal and Bioscience Research Department, Teagasc, Animal and Grassland Research and Innovation CentreMoorepark, Fermoy, Co. Cork, Ireland; ^4^Irish Cattle Breeding FederationBandon, Co. Cork, Ireland; ^5^Molecular Population Genetics, Smurfit Institute of Genetics, Trinity College DublinDublin 2, Ireland

**Keywords:** GWAS, mastitis, SNP, somatic cell score, Holstein-Friesian

## Abstract

Mastitis is an inflammation-driven disease of the bovine mammary gland that occurs in response to physical damage or infection and is one of the most costly production-related diseases in the dairy industry worldwide. We performed a genome-wide association study (GWAS) to identify genetic loci associated with somatic cell score (SCS), an indicator trait of mammary gland inflammation. A total of 702 Holstein-Friesian bulls were genotyped for 777,962 single nucleotide polymorphisms (SNPs) and associated with SCS phenotypes. The SCS phenotypes were expressed as daughter yield deviations (DYD) based on a large number of progeny performance records. A total of 138 SNPs on 15 different chromosomes reached genome-wide significance (corrected *p*-value ≤ 0.05) for association with SCS (after correction for multiple testing). We defined 28 distinct QTL regions and a number of candidate genes located in these QTL regions were identified. The most significant association (*p*-value = 1.70 × 10^−7^) was observed on chromosome 6. This QTL had no known genes annotated within it, however, the Ensembl Genome Browser predicted the presence of a small non-coding RNA (a Y RNA gene) in this genomic region. This Y RNA gene was 99% identical to human RNY4. Y RNAs are a rare type of non-coding RNA that were originally discovered due to their association with the autoimmune disease, systemic lupus erythematosus. Examining small-RNA sequencing (RNAseq) data being generated by us in multiple different mastitis-pathogen challenged cell-types has revealed that this Y RNA is expressed (but not differentially expressed) in these cells. Other QTL regions identified in this study also encoded strong candidate genes for mastitis susceptibility. A QTL region on chromosome 13, for example, was found to contain a cluster of β-defensin genes, a gene family with known roles in innate immunity. Due to the increased SNP density, this study also refined the boundaries for several known QTL for SCS and mastitis.

## Introduction

Mastitis is an inflammatory disease of the bovine mammary gland that occurs in response to physical damage or infection with pathogenic microorganisms such as *Escherichia coli*, *Streptococcus uberis*, and *Staphylococcus aureus* (Schukken et al., 2011). Upon infection of the mammary gland, macrophages and epithelial cells release cytokines which cause polymorphonuclear neutrophils (PMNs), monocytes, and other leukocytes to migrate from the blood to the site of infection in the mammary tissue (Paape et al., 2003). This influx of leukocytes results in an increased level of what is commonly termed “somatic cells” in the milk. Clinical mastitis (CM) is common in dairy herds and is estimated to occur in 20–40% of cows in a dairy herd annually (Heringstad et al., 2000). The costs associated with mastitis are high with mastitis-related costs in the EU estimated at €1.55 billion annually (http://www.sabre-eu.eu/). The frequency and cost of mastitis, along with increased public interest in animal welfare, and concerns regarding the use of antibiotics in livestock, ensure that mastitis is one of the most important production diseases in the dairy industry and therefore any method to reduce the incidence of mastitis would be welcomed. CM has a heritability of 0.03 which means the response to selection will be proportionally small; however, selection for resistance to mastitis is still possible (Heringstad et al., 2000; Carlen et al., 2004). Obtaining direct measures of CM for a sufficiently large number of progeny can, however, be difficult and in the recent past, CM incidence has only been recorded routinely in some Nordic countries (Heringstad et al., 2000). In Ireland and in many other countries, national dairy industries routinely record somatic cell count (SCC) as an indicator trait. As mentioned previously, the host response to intra-mammary infection results in an influx of somatic cells into the milk. The SCC phenotype measures the concentration (cells/ml) of these cells in a milk sample and therefore acts as an indicator of intra-mammary infection. The log transformation of SCC, called somatic cell score (SCS), exhibits a strong genetic correlation (~0.70) with CM (Rupp and Boichard, 2003; Bloemhof et al., 2009) and with a heritability of ~ 0.15 (Mrode and Swanson, 1996) it is a useful phenotype which has been used in a number of association studies investigating the genetic basis of mastitis resistance (Ashwell et al., 1998; Heyen et al., 1999).

The advent of high-throughput genotyping arrays has enabled researchers to perform association studies on a genome-wide scale. A number of genome-wide association studies (GWAS) have detected QTL for SCS in cattle and these are concentrated on chromosomes 5, 8, 11, 18, and 23 (Khatkar et al., 2004; Smaragdov, 2006). These associations help us to better understand the mechanisms underlying mastitis resistance, to identify likely candidate genes and Illuminate the underlying complex nature of the SCS phenotype. An additional role for these QTL regions may be to provide prior information to statistical models used in the selection of animals for breeding. A recent study tested this hypothesis and found that inclusion of QTL as prior information could lead to increased accuracy of selection in some traits (Brøndum et al., 2012).

When designing a GWAS there are two important factors, amongst others (e.g., effect size), which may affect the statistical power of that study to detect associations: (1) the number of markers genotyped and (2) the number of samples which have been genotyped. The GWAS approach relies on the concept that markers are in linkage disequilibrium (LD) with the causal variant and greater LD between the two results in greater power to detect the association (Spencer et al., 2009). In this regard it has been observed that genotyping larger number of SNPs through the use of higher density genotyping arrays can increase the power of the resultant GWAS (Spencer et al., 2009). A variety of genotyping arrays are available in cattle which differ in the number of SNPs which they can genotype ranging from low density (~7,000 SNPs) to medium density (~54,000 SNPs) and upwards to high density (~780,000 SNPs). In general, recent GWAS in Holstein-Friesian dairy cattle have utilized genotyping arrays which assay ~54,000 SNPs (Cole et al., 2009; Pryce et al., 2010; Meredith et al., 2012). This is reasonable because it was estimated that only ~50,000 SNPs would be required to achieve sufficient LD (*r*^2^ ≥ 0.2) for a GWAS in Holstein-Friesian cattle due to the extensive LD in the breed (de Roos et al., 2008). However, as discussed by Khatkar et al. (2008), increased SNP density should lead to higher-powered GWAS due to the stronger average LD between SNPs and coverage achieved across the genome. Therefore, the use a higher-density genotyping array in the form of the Illumina High-Density Bovine BeadChip (Illumina Inc., San Diego, CA, USA) paves the way for such a GWAS to be performed.

The objective of this study was to perform a GWAS using a group of 702 Holstein-Friesian bulls which have been genotyped using the Illumina High-density Bovine BeadChip (777,962 SNPs) to detect associations with high-reliability SCS phenotypes, estimated using a large number of progeny performance records. We identified a number of QTL regions and genes within and around these regions which may play a role in the SCS trait, and by extension resistance to mastitis.

## Materials and methods

### DNA extraction

Thawed frozen semen was washed twice in phosphate-buffered saline (pH 7.4), and cell pellets were harvested via centrifugation and re-suspended in 450 μL of pre-warmed extraction buffer (10 mM Tris, pH 8.0; 10 mM EDTA, pH 8.0; 1% SDS; 100 mM NaCl); 15 μL of 2-mercaptoethanol was added. Samples were incubated at 55°C for 15 min followed by the addition of 10 μL of proteinase K (20 mg/mL). Lysis occurred following overnight incubation at 60°C. DNA was then extracted using the Maxwell instrument (Promega Corp., Madison, WI) according to the manufacturer's instructions. Genotyping using the Illumina High-density Bovine BeadChip (Illumina Inc., San Diego, CA) and subsequent genotype calling were carried out by Geneseek (Lincoln, NE).

### Illumina high-density bovine genotypes

Seven hundred and forty-seven Holstein-Friesian sires were genotyped (777,962 SNPs) on the Illumina High-density Bovine BeadChip. SNP positions were based on the UMD 3.1 bovine genome assembly (Zimin et al., 2009). The genotype data underwent a series of quality control checks and a number of animals and SNPs were removed in the following order; (1) 8 animals and 14,730 SNPs were removed which exhibited over 5% Mendelian inconsistencies (2) 15 animals and 10,412 SNPs had greater than 10% missing SNP calls and were removed, (3) 40 SNPs were removed due the proportion of heterozygous genotypes being >90%, (4) 6,409 SNPs violating Hardy-Weinberg equilibrium (*p*-value <0.000001) were removed and (5) 3,153 SNPs located in mitochondrial DNA, on the Y chromosome or which had an unknown genomic position were removed. A further 165,037 SNPs were removed before association analysis due to a minor allele frequency <0.05; of these, 97,325 SNPs were monomorphic. An additional 22 animals were removed from the association analysis that did not have a daughter yield deviation (DYD) phenotype for SCS. Following all of these edits, a group of 702 Holstein-Friesian sires genotyped at 578,181 SNPs was available for association testing.

### Daughter yield deviations for somatic cell score

The phenotypes for SCS were expressed as DYD and were based on genetic evaluations carried out in January 2012 by the Irish Cattle Breeding Federation (ICBF). The DYD phenotypes are genetic evaluations undertaken using the performance records from an animal's own progeny. The bulls in this study were widely-used for artificial insemination in Ireland and hence have a large number of progeny performance records. Therefore, animal phenotypes are not just based on a single measure of a trait but instead on a composite of all their progeny performance data. These DYD phenotypes for average SCS are estimated in Ireland using a repeatability animal model across the first five lactations according to the protocol of Berry et al. (2007). The reliability of a phenotype is a measure of the amount of information used in the genetic evaluation of that animal. The animals in this study were bulls widely-used for artificial insemination in Ireland and so they have a large number of progeny and therefore high phenotypic reliabilities. Reliabilities range from 0-1 with zero being the lowest reliability possible. Animals with high reliability (0.99) have a large number of progeny (~1200 + progeny) and the reliability drops as the number of progeny (and hence performance records) decreases (e.g., a reliability of 0.60 equates to an animal with ~30 progeny). Sires in this study had a mean (*S.D.*) reliability of 0.88 (0.13) which is roughly equivalent to an animal with 150 progeny. The SCS phenotype was checked for normality using the R statistical software (R Core Team, 2012). A pedigree file, containing at least four generations of the genotyped animal's ancestors, was created for the 702 animals resulting in a pedigree file with 4,739 animals.

### Statistical analysis

Associations (702 animals genotyped at 578,181 SNPs) were quantified using a single SNP regression approach. The dependent variable (i.e., the respective trait expressed as a DYD) was regressed on each SNP individually in a mixed animal model fitted in DMU (Madsen and Jensen, 2010). Each SNP was fitted as a fixed, continuous effect. The individual animal was fitted as a random effect with relationships among animals accounted for using the additive relationship matrix based on pedigree information. Correction for multiple testing was performed using the Benjamini-Hochberg procedure (Benjamini and Hochberg, 1995) as implemented by the “p.adjust” function in version 2.14.1 of the R statistical package. A genome-wide significance threshold was set as a corrected *p*-value ≤ 0.05 which equated to an uncorrected *p*-value of ~1.19 × 10^−5^.

### Defining QTL regions based on local LD structure

A quantitative trait locus (QTL) region surrounding a detected significant association was defined based on the local LD structure for each SNP, individually. Pairwise LD, as measured by *r*^2^, between all SNPs within 5 Mb upstream and downstream of a significant SNP was calculated using PLINK (Purcell et al., 2007). This allowed us to graphically examine the LD structure surrounding each significant SNP. Within this 10 Mb region (5 Mb upstream and downstream) a cut-off of *r*^2^ ≥ 0.5 was used and the SNP with this level of LD which were furthest upstream or downstream of the significant SNP were noted, thereby forming a QTL region. Any adjacent QTL regions which overlapped were combined into a single QTL region using the maximum and minimum genomic positions as boundaries; this was carried out in R/Bioconductor (Gentleman et al., 2004) using the reduce function in the “*GenomicRanges*” package (Aboyoun et al., 2010). In this way, the significant SNPs were used to identify a list of QTL regions. These regions were subsequently investigated to ascertain which, if any, genes were located in each region.

## Results

### Sample and SNP summary statistics

The SCS phenotypes were normally distributed with a mean (*S.D.*) of 36.57 (104.10) with minimum and maximum values of −236 and 560, respectively. The 702 animals had a mean (*S.D.*) SCS phenotype reliability of 0.88 (0.13). The mean distance between SNPs across all the chromosomes was 6,174 bp but was lower when only considering the autosomes (4,342 bp).

### Genome-wide associations

**Figure 1:**
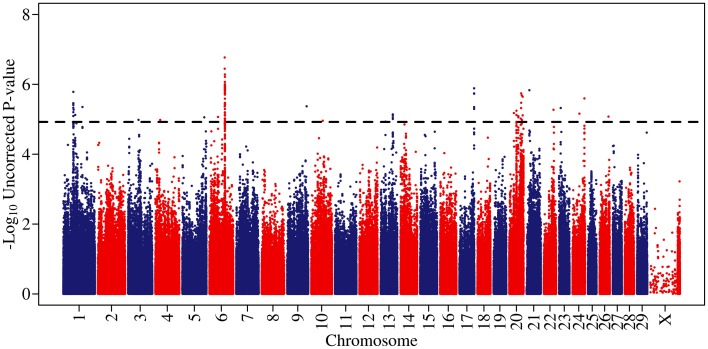
**A Manhattan plot of genome-wide associations for somatic cell score in 702 Holstein-Friesian sires.** The −log_10_ of the uncorrected *p*-values is shown for each SNP (y-axis). The genome-wide significance threshold is indicated by a dashed line.

Genome-wide associations were estimated using the 702 animals at 578,181 SNPs and estimates of SNP effect, standard error, t statistic, uncorrected and corrected *p*-values for all SNPs can be viewed in Supplementary Table 1. Twenty eight distinct QTL regions (Table 1) were significantly associated with SCS, with 138 SNPs exceeding the threshold for genome-wide significance after correction for multiple testing. The significantly associated SNPs were located across 15 different chromosomes (chromosomes 1, 3, 4, 5, 6, 9, 10, 13, 17, 20, 21, 22, 23, 24, 25, and 26) (Figure 1). These QTL regions were cross-referenced to known QTLs for SCS / SCC and CM in the cattle QTL database (http://www.animalgenome.org/QTLdb/cattle.html) as of May 2013 and 11 QTLs fell within previously identified QTL regions; this left 17 remaining novel QTL regions. In general, the QTL regions identified using the high-density chip were more strongly associated with SCS and the boundaries of the QTL regions were more precise than those identified in our previous SCS GWAS in a similar population of animals using the lower-density Illumina BovineSNP50 BeadChip (Meredith et al., 2012).

**Table 1:** **QTL regions identified for SCS**.

**Chr**	**QTL start (bp)**	**QTL end (bp)**	**QTL length (bp)**	**No. of SNPs**	**No. of Genes**	Gene names^*^
1	49,885,358	50,452,539	567,182	10	1	ALCAM
1	51,679,722	51,695,056	15,335	1	0	
1	59,651,839	59,799,918	148,080	1	1	ZBTB20
1	96,026,399	96,600,720	574,322	1	3	FNDC3B
						TMEM212
						PRKCSH
3	52,269,691	52,300,879	31,189	1	0	
4	24,799,926	24,800,412	487	1	1	ISPD
5	110,275,795	110,275,795	1	1	1	SOX10
6	43,203,039	43,259,077	56,039	1	0	
6	76,649,555	77,943,085	1,293,531	82	1	Y_RNA
9	97,459,242	97,459,242	1	1	1	WTAP
10	57,715,416	57,715,416	1	1	1	BT.37181
13	24,548,898	24,720,132	171,235	1	2	C10ORF67
						OTUD1
13	61,182,238	61,619,701	437,464	7	13	TRIB3NRSN2
						ENSBTAG0000
						0027386
						C20ORF96
						DEFB129
						DEFB119
						DEFB122A
						DEFB122
						BT.54136
						BT.100199
						REM1
						ZCCHC3
						DEFB117
17	73,874,968	73,891,077	16,110	6	1	ENSBTAG0000
						0023007
20	36,064,784	36,222,775	157,992	4	1	EGFLAM
20	45,581,169	45,638,063	56,895	4	0	
20	57,650,372	57,746,837	96,466	1	0	
20	59,314,402	59,314,402	1	1	1	DNAH5
20	59,848,168	59,899,899	51,732	2	0	
20	62,174,229	63,901,891	1,727,663	2	13	CTNND2 DAP
						ANKRD33B
						BT.22715
						MARCH6
						ENSBTAG0000
						0022809
						CMBL
						BT.68634
						BT.65753
						BT.76062
						U2
						SNORD123
						U6
20	67,038,772	67,654,036	615,265	2	0	
20	23,201,811	23,616,460	414,650	1	5	BT.66498
						IL31RA
						BT.35994
						SLC38A9
						SNORA18
21	13,978,417	14,022,698	44,282	1	0	
22	48,483,717	48,603,179	119,463	1	2	BT.103068
						TMEM110
23	8,449,971	8,449,971	1	1	0	
24	34,723,910	34,723,910	1	1	0	
24	59,670,860	59,759,141	88,282	1	1	MC4R
26	45,116,066	45,149,850	33785	1	0	

The Ensembl gene identifier is provided where a gene name was not available.

**Figure 2:**
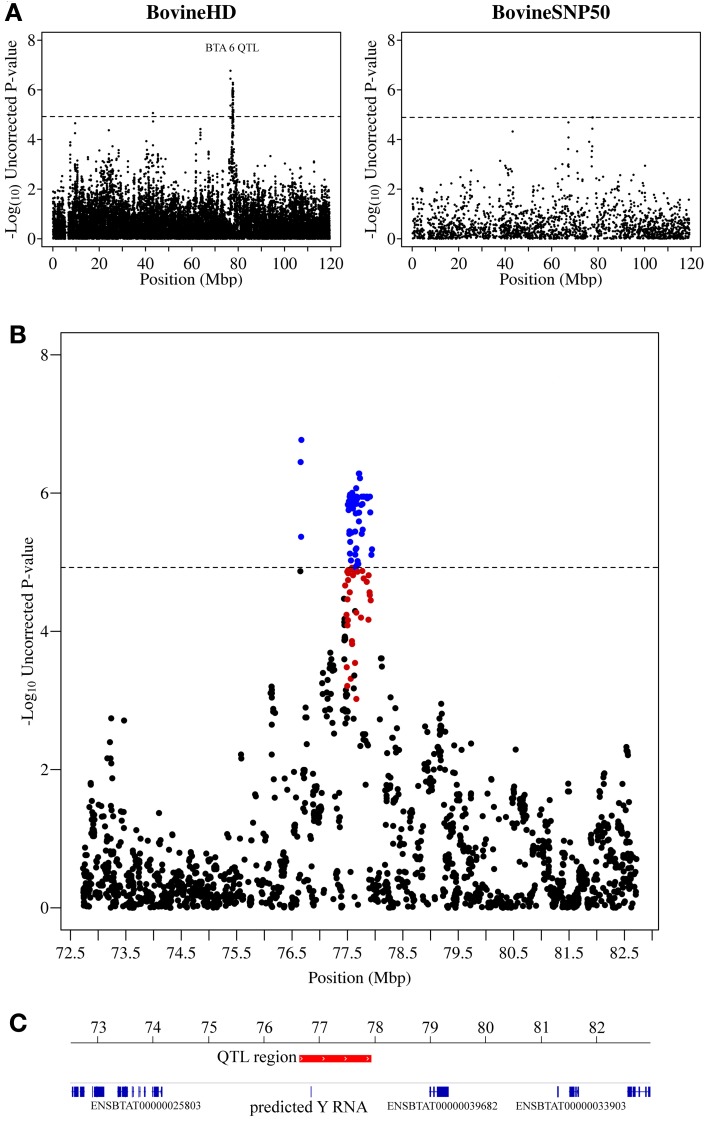
**QTL region associated with SCS on chromosome 6. (A)** Compares the associations from this study (Illumina BovineHD BeadChip) with those of a previous study (Meredith et al., 2012) of similar design but using a lower-density genotyping array (Illumina BovineSNP50 BeadChip). **(B)** Shows the associated region (associations from current study) in closer detail with significant SNPs indicated in blue and any SNP in moderate to strong linkage disequilibrium (*r*^2^ ≥ 0.5) with a significant SNP indicated in red. **(C)** Shows the genes that are annotated in this region based on the UMD 3.1 assembly annotation from Ensembl. The genome-wide significance threshold is indicated by a dashed line.

The strongest association signal in this study was detected on chromosome 6 (located at 76.6–77.9 Mb) with 82 SNPs exceeding the genome-wide significance threshold. The most significant SNP had a *p*-value of 1.70 × 10^−7^ (Figure 2). No known genes were annotated in this region, however, there was a predicted Y RNA gene (ENSBTAG00000043492), which was located 188,460 bp downstream of the most significant SNP (BovineHD0600021283). Y RNAs are a rare type of small non-coding RNA which were first detected in the investigation of the autoimmune disease systemic lupus erythematosus (Lerner et al., 1981). Y RNAs are evolutionarily conserved (Mosig et al., 2007) and this bovine Y RNA was found to be 99% identical to human Y RNA 4 (RNY4). Little is currently known regarding the function of Y RNAs, except that they are components of Ro ribonucleoproteins (RNPs) (Verhagen and Pruijn, 2011) and may have a role in RNA quality control and in the initiation of DNA replication (Krude et al., 2009).

Examination of small RNAseq data recently published by our group (Lawless et al., 2013) showed that this bovine predicted Y RNA was in fact a real expressed gene and was expressed [albeit at a relatively low level—average 12 reads per million (RPM)] in bovine mammary epithelial (BME) cells infected with a mastitis causing pathogen, *Streptococcus uberis*. This Y RNA did not, however, appear to be differentially expressed in BMEs in comparison to uninfected controls. There is no evidence to link differential expression of this Y RNA gene to mastitis and so we hypothesize that SNPs may occur within the Y RNA which could lead to structural differences in the Y RNA, affecting its function. Unfortunately, no SNP genotyped by the Illumina bovine high-density BeadChip was located within this gene.

**Figure 3:**
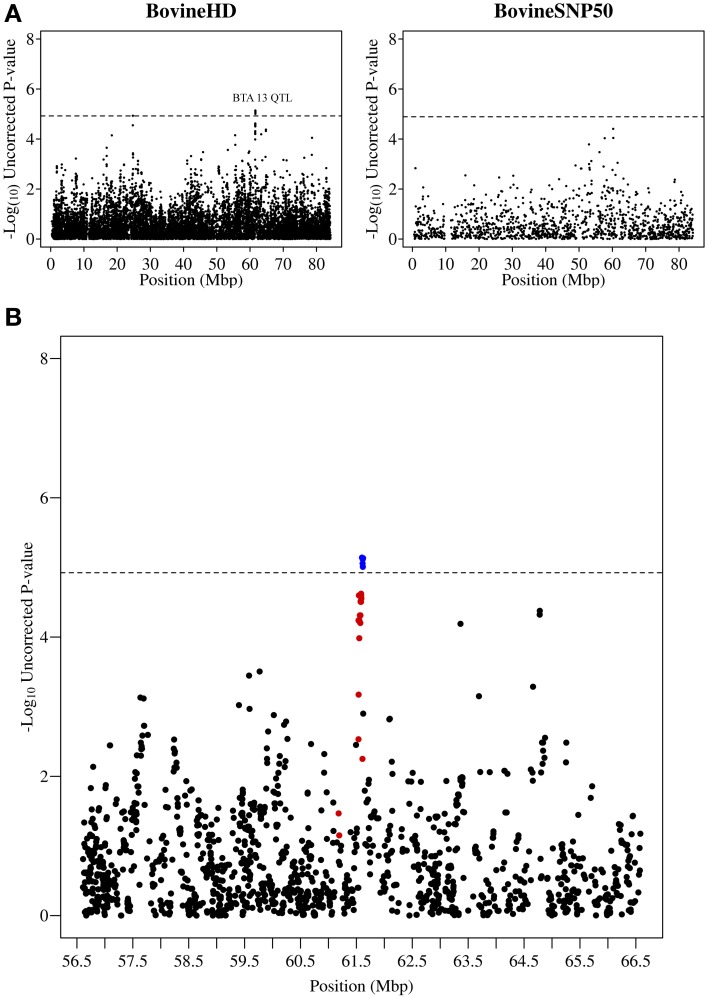
**QTL region associated with SCS on chromosome 13. (A)** Compares the associations from this study (Illumina BovineHD BeadChip) with those of a previous study (Meredith et al., 2012) of similar design but using a lower-density genotyping array (Illumina BovineSNP50 BeadChip). **(B)** Shows the associated region (associations from current study) in closer detail with significant SNPs indicated in blue and any SNP in moderate to strong linkage disequilibrium (*r*^2^ ≥ 0.5) with a significant SNP indicated in red. The genome-wide significance threshold is indicated by a dashed line.

Other QTL regions identified in this study also contained strong candidate genes for mastitis susceptibility. A significant association was detected on chromosome 13 at 61.2–61.6 Mb with the most significant SNP having a *p*-value of 7.23 × 10^−6^ (Figure 3). The association signal was observed to be a more precise and significant signal than had been identified in our previous 50K GWAS study (Meredith et al., 2012). This QTL region, which extends for 437 kb, overlaps a known cluster of β-defensin genes (Cormican et al., 2008) which encompasses five β-defensin genes (*DEFB129, DEFB119, DEFB122A, DEFB122, DEFB117*) and two genes for β-defensin precursors (*BT*.*54136, BT*.*100199*). β-defensins are a family of host defence peptides involved in the innate immune response and which are found across numerous species including cattle, humans and chickens (Ganz, 2003).

**Figure 4:**
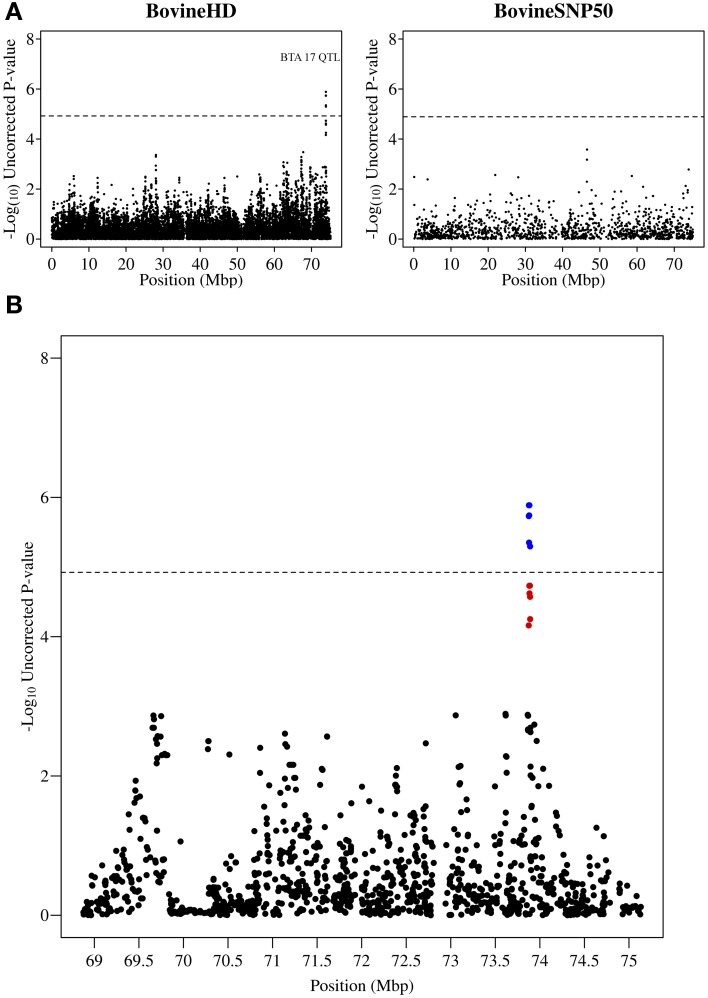
**QTL region associated with SCS on chromosome 17. (A)** Compares the associations from this study (Illumina BovineHD BeadChip) with those of a previous study (Meredith et al., 2012) of similar design but using a lower-density genotyping array (Illumina BovineSNP50 BeadChip). **(B)** Shows the associated region (associations from current study) in closer detail with significant SNPs indicated in blue and any SNP in moderate to strong linkage disequilibrium (*r*^2^ ≥ 0.5) with a significant SNP indicated in red. The genome-wide significance threshold is indicated by a dashed line.

A peak of six significantly associated SNPs was also detected near the end of chromosome 17 at 73.87–73.89 Mb with the most significant of these SNPs having an uncorrected *p*-value of 1.3 × 10^−6^ (Figure 4). A strong candidate gene, Mitogen-activated protein kinase 1 (MAPK1), which has been shown to be significantly down-regulated in bovine primary BME cells in response to challenge with *Escherichia coli* (Gunther et al., 2011), was found to be located 120 kb downstream of the detected QTL region.

A significant association signal was also detected on chromosome 1 with a QTL region extending from 49.9–50.5 Mb; the most significant SNP (BovineHD0100014076) in this region had an uncorrected *p*-value of 1.64 × 10^−6^. Only one gene, activated leukocyte cell adhesion molecule (*ALCAM*), overlapped with this QTL region.

On chromosome 20, a number of significantly associated QTL regions were detected. One of these extended from 23.2–23.6 Mb (the most significant SNP in this region was BovineHD2000007011—uncorrected *p*-value = 6.64 × 10^−6^). One gene of interest in this region, which contained a significantly-associated SNP, was interleukin-31 receptor subunit alpha precursor (*IL31Rα*) gene. Another QTL region of interest on chromosome 20 was identified from 62.2–63.9 Mb with the most associated SNP, BovineHD2000017761, having an uncorrected *p*-value of 2.08 × 10^−6^. This region was gene-rich with a total of 13 bovine genes located within its boundaries. In addition, another QTL region on chromosome 20 located 36.0–36.2 Mb was detected which had a number of nearby adjacent complement genes (*C9, C6, BT*.*69954*, and *C1QTNF3*). These complement genes are part of the complement system, a collection of serum proteins and cell surface receptors, which form an integral part of the innate immune system that are activated via host pattern-recognition receptors which recognize pathogen-associated molecular patterns (PAMPs) on pathogenic microorganisms (Medzhitov and Janeway, 2002; Medzhitov, 2007). The complement system is involved in innate immune responses such as inflammation, opsonization, chemotaxis of phagocytes to the site of infection, and cell lysis, and also is believed to play a role in adaptive immunity via the regulation of B and T cell response (Carroll, 2004; Medzhitov, 2007). Therefore, these complement genes are good candidate genes to be involved in resistance to mastitis.

Finally, a QTL region detected on chromosome 26 extending from 45.12–45.15 Mb fell within a larger QTL region previously associated with SCS (Zhang et al., 1998) and CM (Lund et al., 2008). However, our detected QTL is much narrower than those previously identified.

### Imputation

Imputation of lower-density (BovineSNP50) genotypes from 525 animals was also carried out using high-density genotypes from the 724 animals used in our study as a reference using the BEAGLE software (Browning and Browning, 2009) (Materials and Methods in Supplementary Document 1). The average imputation accuracy for the animals was 0.98 (ranging from 0.79–0.99). SNPs had an average imputation accuracy of 0.98 (ranging from 0.47–1.00). We repeated our GWAS using a combined dataset of both the high-density genotyped and imputed datasets (1,227 animals). The associations that were detected in the original GWAS failed to reach genome-wide significance in the combined dataset. As discussed below this may be due to a loss of power when including the imputed data.

## Discussion

This study identified a large number of SNPs significantly associated with SCS, a trait that is highly correlated with bovine mastitis, one of the most costly production diseases in dairy cattle. To the best of our knowledge this study is the first to use the Illumina BovineHD BeadChip to undertake a GWAS for SCS. The substantially increased SNP density of the BovineHD platform (1 SNP/~4,500 bp vs. 1 SNP/~62,000 bp for the BovineSNP50) appears to have provided increased resolution and power to detect QTL associated with SCS in this study (see Figures 2–4). Furthermore, this study enabled more precise mapping of both previously identified and novel QTL due to the increased SNP density. Twenty eight distinct QTL regions were defined and a number of promising candidate genes located in these QTL regions were identified.

The strongest association with SCS in our study was identified on chromosome 6. The only gene that appears to be located within this QTL encodes a rare type small non-coding RNA called a Y RNA. We have found that this Y RNA gene is 99% identical to human Y RNA 4 (RNY4) and was expressed but not differentially expressed in BME cells infected with a mastitis causing pathogens (Lawless et al., 2013). Further work is needed to characterize this gene and determine whether it plays a role in susceptibility to mastitis. Several previous studies have also implicated regions of chromosome 6 as being associated with SCS or mastitis, although none have identified the same QTL as uncovered in this study. Bennewitz et al., for example used microsatellite markers to identify a large QTL (~108 Mb) on BTA6 (Bennewitz et al., 2004). The size of the QTL, however, made it impossible to pinpoint particular candidate genes that might be associated with the trait. An association with CM near the casein gene complex at ~90 Mb on chromosome 6 has also been identified (Nilsen et al., 2009; Sodeland et al., 2011). This region was not significantly associated with SCS in our study, however. This may be explained by differences in the phenotypes used in the association study. Recently, Sahana et al. (2013) found a region on chromosome 6 extending from 83-93 Mb associated with CM which is closer to our detected QTL region; however, the association detected in our study is still ~6 Mb away from this region and so can be considered a distinct QTL region. A QTL region associated with SCS located at 78.7 Mb on BTA6 was also detected in a previous study of Irish Holstein-Friesian cattle (Meredith et al., 2012).

Our study also detected a novel QTL region located at 61.1–61.6 Mb on chromosome 13 associated with SCS. Previous studies have identified genetic markers associated with SCS nearby on chromosome 13 at 57.5–58.5 Mb (Lund et al., 2008; Sahana et al., 2013) but none of them overlap our associations. The QTL region in this study overlaps a well-known cluster of β-defensin genes and these genes, known for their host defence function, would appear to be strong candidate genes for having a role in mastitis. β-defensin genes have also been shown to be expressed in bovine udder tissue in response to infection (Goldammer et al., 2004; Swanson et al., 2004) and expression levels of particular β-defensins have been shown to be positively correlated with SCS (Swanson et al., 2004). In addition, β-defensins are also believed to influence chemotaxis of white blood cells to the site of infection (Kaiser and Diamond, 2000; Ganz, 2003) which provides another mechanism by which these genes may play an important functional role in mastitis resistance in the dairy cow.

The strong, novel associations detected on chromosome 17 provided a number of other candidate genes which were in close proximity to the QTL region. The *MAPK1* gene located within 125 kb of this QTL is a member of the MAPK family which is a key pathway involved in the recruitment of leukocytes to the site of infection (Herlaar and Brown, 1999; Kaminska, 2005). However, a number of other nearby genes also have merit including (1) immunoglobulin lambda-like polypeptide 1 precursor (*IGLL-1*) which has been observed to have increased expression in bovine udder tissue in response to *Streptococcus uberis* challenge (Swanson et al., 2009), (2) Synaptosomal-associated protein 29 (*SNAP29*) whose increased expression has been linked to enhanced internalization and killing of *E*.* coli* by mast cells (Wesolowski et al., 2012), (3) Stromal cell-derived factor 2-like protein 1 (*SDF2L1*) which is involved in the processing of a wide range of defensins and (4) mir301b, a microRNA which has been shown to be significantly down-regulated in response to lipopolysaccharide (LPS) (Zheng et al., 2012).

A number of separate strongly associated QTL regions were identified on chromosome 20 in this study. Several studies have reported associations with SCS/CM with genetic markers on chromosome 20 (Sodeland et al., 2011; Meredith et al., 2012; Sahana et al., 2013) with many of them overlapping or nearby the regions detected in this study. Nevertheless, a number of possible candidate genes have been identified including the *IL31Rα* gene which acts as part of a receptor complex for the *IL-31* cytokine in the activation of signaling pathways including the *JAK-STAT* and *MAPK* pathways which can initiate a wide range of immunological processes (Zhang et al., 2008; Cornelissen et al., 2012). The detection of a QTL region in close proximity to a number of genes of the complement system also looks promising given the importance of the complement system in both host innate and adaptive immunity.

The associated region on chromosome 1 from 49.9–50.5 Mb contained a single gene called *ALCAM*. This gene, also known as *CD166 antigen*, is a transmembrane receptor which has been examined extensively in human cancer research and has been implicated in leukocyte adhesion/migration and T cell activation (Hassan et al., 2004; Zimmerman et al., 2006; Cayrol et al., 2007). In addition, in a study of milk somatic cells which had been challenged with *Staphylococcus epidermidis* or *Staphylococcus aureus*, *ALCAM* was shown to be over-expressed in the milk somatic cells of a mastitis-resistant line of sheep in comparison with a mastitis-susceptible line (Bonnefont et al., 2011). Therefore, *ALCAM* seems a plausible candidate gene to be involved in mastitis resistance.

Statistical power is a key issue in any GWAS and we endeavored to improve our study power via imputation of ungenotyped markers in additional animals. However, the associations were not significant on a genome-wide scale when using the combined dataset with the imputed genotypes. There are several possible reasons which may underlie this result. The imputation accuracy observed across all animals was high (0.98), however, a number of animals had an imputation accuracy score <0.90 with one animal having an accuracy score of 0.79. This variability in imputation accuracy may have occurred due to several reasons. Firstly, it is possible the size of our reference panel (*n* = 724) is inadequate to accurately impute ungenotyped SNPs for animals genotyped on the Bovine SNP50 chip leading to a loss certainty of imputed genotypes. The accuracy of imputation can vary widely for a particular SNP depending on the surrounding LD structure (de Bakker et al., 2008) and this may have contributed to non-uniform imputation accuracy. Huang et al. (2009) looked at the relationship between the accuracy of imputation and the power of a subsequent association study using imputed markers. They found that even small decreases in the imputation accuracy can lead to large reductions in the power of GWAS and that a substantial increase in sample size would be required to maintain the same level of power. Given the variability in imputation accuracy that we observed in our study, this reduction in power due to imperfect imputation may explain the lack of associations with genome-wide significance observed when including the imputed animal in the association dataset.

## Conclusions

The use of the Illumina High-density BeadChip afforded our study a SNP density and resolution far above that previously reported for a GWAS for SCS in dairy cattle. We have identified 138 SNPs significantly associated with SCS which make up 28 distinct QTL regions across 15 chromosomes. Several of these QTL regions are novel associations. Additionally, the increased SNP density in our study allowed us to more precisely map QTL intervals with some previously known QTL intervals being narrowed. Many of these strongly associated QTL regions encompass promising candidate genes such as the β-defensins on chromosome 13 and a Y RNA gene on chromosome 6. These QTL regions and their candidate genes can form the basis of more detailed research into the genetic mechanisms that underlie resistance to mastitis in the dairy cow.

## Author contributions

Experimental design was carried out by Brian K. Meredith, David J. Lynn, Daniel G. Bradley and Donagh P. Berry. DNA extraction was carried out by Emma K. Finlay. Phenotypes were provided by Donagh P. Berry and Francis Kearney. Analysis was undertaken by Brian K. Meredith and David J. Lynn. Manuscript preparation was carried out by Brian K. Meredith, David J. Lynn, Daniel G. Bradley, Donagh P. Berry and Alan G. Fahey.

## Conflict of interest statement

The authors declare that the research was conducted in the absence of any commercial or financial relationships that could be construed as a potential conflict of interest.
