# Supplementary material for: A genome-wide association study for somatic cell score using the Illumina high-density bovine beadchip identifies several novel QTL potentially related to mastitis susceptibility
Source: Front Genet. 2013 Nov 6;4:229. doi: 10.3389/fgene.2013.00229 (PMC3818585; doi:10.3389/fgene.2013.00229)
Supplement: Supplementary file 2 [file DataSheet2.DOC]

**Supplementary Document 1**

**Illumina bovine SNP50 genotypes**

Further to the high-density genotypes described above, 542 additional other Holstein-Friesian sires were genotyped at 54,001 SNPs using the Illumina Bovine SNP50 BeadChip (Illumina Inc., San Diego, CA). The same DNA extraction protocol as described previously was used to extract DNA from semen samples. SNP positions were based UMD 3.1 bovine genome assembly. A total of 724 of the aforementioned genotyped on the high-density chip were used as reference animals for imputation. These 724 animals each had 743,218 SNPs which passed quality control measures before imputation (minor allele frequency measure was not applied for imputation datasets) and SNPs on the SNP50 BeadChip (54,001 SNPs) that were not present in the high-density, quality-controlled SNP set (743,218 SNPs) were removed which left 48,734 (46,138) remaining SNPs. A series of genotypic edits were then applied to the 542 animals and 48,734 SNPs. Mendelian inconsistencies between parent-offspring pairs were identified using a larger dataset of 2,618 Holstein-Friesian animals with SNP50 genotypes which included the 542 animals used here. A number of animals and SNPs were removed as a result of the following edits; 1) 17 animals and 39 SNPs were removed which exhibited over 5% mendelian inconsistencies, 2) zero animals and 303 SNPs were removed due to having greater than 10% missing SNP calls, 4) zero SNPs were removed due the proportion of heterozygous genotypes being greater then 90% and 5) 46 SNPs violating Hardy-Weinberg equilibrium (p-value <0.000001) were removed. Therefore, 525 animals genotyped at 45,750 SNPs were used for the imputation step.

**Daughter yield deviations for somatic cell score**

The somatic cell score phenotypes for these animals were calculated in the same way as previously described for the high-density animals and therefore were also expressed as DYDs. The SCS phenotype was checked for normality using a Q-Q plot in the R statistical software. All 525 animals which passed genotypic edits had a somatic cell score phenotype. After imputation these 525 animals were combined with the 702 high-density animals to create a combined dataset of 1,227 animals genotyped at 578,181 SNPs. A pedigree file, containing at least four generations of the genotyped animal’s ancestors, was created for these 1,227 animals resulting in a pedigree file with 7,860 animals. Therefore, after imputation, associations were carried out using 1,227 animals each genotyped at 578,181 SNPS.

**Imputation protocol**

Imputation was carried out using BEAGLE v3.3.2 (Browning and Browning, 2009) with known parent-offspring pairs (n=721 as 591 pairs) included as pairs and the remaining animals (n=528) treated as unrelated. The accuracy of imputation was estimated using genotypes for chromosome 6; 50 randomly chosen high-density animals were selected and high density SNPs of these animals which are not on the Illumina BovineSNP50 SNP manifest were masked. The imputation was carried out and imputed genotypes were then compared with the known, real genotypes to check the accuracy of imputation for both SNPs and animals, respectively. This process was repeated 30 times to provide an estimate of imputation accuracy for animals and SNPs, respectively. Animals which appeared several times in the imputation runs had their accuracy scores averaged to give a single score for that animal; SNP accuracy scores were averaged across all 30 imputation runs.
